# Colorectal cancer screening among Hispanics/Latinos in the HCHS/SOL sociocultural ancillary study

**DOI:** 10.1016/j.pmedr.2019.100947

**Published:** 2019-07-04

**Authors:** Sheila F. Castañeda, Linda C. Gallo, Jesse Nodora, Gregory A. Talavera, Frank J. Penedo, Kelly R. Evenson, Maria Lopez-Gurrola, Sylvia Smoller-Wassertheil, Lisa A.P. Sanchez-Johnsen, Patricia Gonzalez, Krista M. Perreira, Samir Gupta

**Affiliations:** a San Diego State University, Graduate School of Public Health, San Diego, CA, United States of America; b University of California, San Diego, Moores Cancer Center, La Jolla, CA, United States of America; c Northwestern University, Chicago, IL, United States of America; d University of North Carolina – Chapel Hill, Department of Epidemiology, Chapel Hill, NC, United States of America; e Einstein College of Medicine, The Bronx, New York, United States of America; f University of Illinois at Chicago, Chicago, IL, United States of America

**Keywords:** Cancer, Early detection of cancer, Behavioral medicine, Epidemiology

## Abstract

Latino adults are more likely to be diagnosed with colorectal cancer (CRC) at later stages compared to white adults which may be explained by disparities in screening rates. The aim of this study was to examine factors associated with three CRC screening indicators [i.e., 1) any CRC screening ever (via, fecal occult blood test (FOBT), sigmoidoscopy, or colonoscopy); 2) FOBT in last year, 3) sigmoidoscopy/colonoscopy in last 10 years) among US Hispanics/Latinos. We analyzed population-based data collected in 2008–2011 from 2265 adults aged 50–75 from San Diego, Bronx, Miami and Chicago from the Hispanic Community Health Study/Study of Latinos Sociocultural Ancillary Study. Based on the Behavioral Model of Health Services Use, the following correlates of CRC screening were examined: predisposing (i.e., age, education, income, acculturation), enabling (i.e., recent physician visit, insurance, recent mammogram), and need (i.e., health-related quality of life and family/personal history of cancer) factors. Separate logistic regression models were analyzed for the three CRC screening indicators. Enabling factors associated with all CRC screening indicators included: health insurance, a recent physician visit, and a mammogram in the last year (women only). For women, being older, more acculturated (i.e., English language or foreign-born but in the US for 10 or more years), and having a personal history of cancer was associated with at least one CRC screening. Findings suggest that improving access and utilization of care among Hispanics/Latinos may be critical for earlier CRC diagnosis and survival.

## Highlights

• Women were significantly more likely than men to have ever had a colonoscopy.
• For men and women, insurance, a recent physician visit, and cancer history are important correlates of CRC screening.
• Among women, an older age, being more acculturated, and having a recent mammogram were correlates of CRC screening.

## 1 Introduction

In 2009, cancer surpassed heart disease as the leading cause of death for Latinos in the United States (US) (American Cancer Society (ACS), 2012). Colorectal cancer (CRC) is among the most common cancer diagnoses and among the top three causes of cancer mortality among Latinos nationwide (American Cancer Society (ACS), 2012; American Cancer Society (ACS), 2014). Latinos are more likely to exhibit late-stage invasive cancer (National Cancer Institute, 2007), and significantly reduced rates of survival (Vernon et al., 2010; Mandelblatt et al., 2009) than are other ethnic/racial groups (Zambrana et al., 1999). Regular use of CRC screening is associated with early detection (American Cancer Society (ACS), 2014). The 2016 US Preventive Services Task Force (USPSTF) recommends regular CRC screening for all individuals age 50 to 75 years. Screening tests include: high-sensitivity guaiac fecal occult blood testing (FOBT) annually, fecal immunochemical testing (FIT) annually, FIT-DNA every three years, sigmoidoscopy every five years alone or every 10 years with annual FIT, or colonoscopy every 10 years (U.S. Preventive Services Task Force, 2016). Latinos, especially those without health insurance, have lower screening rates than other racial/ethnic groups nationwide (American Cancer Society (ACS), 2012). The 2015 National Health Interview Survey (NHIS) indicate that Latinos (47%) are less likely to be adherent with current CRC screening guidelines as compared to non-Hispanic Whites (64%) or non-Hispanic Blacks (59%) (White et al., 2017). Reasons for lower CRC screening rates among Latinos have been understudied.

This study used the Behavioral Model of Health Services Use (BMHSU) (Babitsch et al., 2012) theoretical framework to explain predictors of CRC screening among Latinos. The BMHSU has been used to examine access and utilization of hospital, dental, and medical care among diverse adults (Andersen et al., 2000; Gelberg et al., 2000; Miller et al., 2008; Stein et al., 2007; Swanson et al., 2003) access and utilization barriers experienced by Latinos (Andersen et al., 1986; Estrada et al., 1990), and cervical, colorectal and breast cancer screening among Latinos (Fernandez and Morales, 2007; Gorin and Heck, 2005). Results using the BMHSU framework suggests that healthcare use is a function of an individual's predisposition to use services (predisposing domain), factors that enable healthcare use (enabling domain), and the need for care (need domain) (Andersen, 1995).

Predisposing factors refer to characteristics that affect the likelihood of healthcare utilization (Stein et al., 2007), and include age, sex, income, education, and acculturation. For example, older Latinas are significantly more likely to obtain mammograms than younger Latinas (Fernandez and Morales, 2007; Gorin and Heck, 2005); however, some studies indicate that age does not significantly relate to breast or cervical cancer screening after adjusting for other factors (Abraido-Lanza et al., 2004). There is also evidence to suggest that Latino males are more likely to have received CRC screening in the past compared to Latina females (Meissner et al., 2006). Lower levels of education and income are associated with lower screening rates among Latinos (Gorin and Heck, 2005; Reyes-Ortiz et al., 2007). The literature offers no consensus on the role of acculturation factors (e.g., language-based acculturation, years in the US, and country of birth) in predicting CRC screening (Fernandez and Morales, 2007; Gorin and Heck, 2005; Otero-Sabogal et al., 2003; Rodriguez et al., 2005; Gonzalez et al., 2012; Savas et al., 2014).

Enabling factors are defined as conditions that make accessing services possible (Andersen, 1995), and include health insurance, access to a regular health care source, and utilization of services (Andersen and Newman, 1973). Studies indicate that having any health insurance (Fernandez and Morales, 2007; Gorin and Heck, 2005; Abraido-Lanza et al., 2004; Carrasquillo and Pati, 2004; De Alba et al., 2005), visiting a physician in the past year (Gorin and Heck, 2005; Frazier et al., 1996), or having a usual source of care (Fernandez and Morales, 2007; De Alba et al., 2005) enables use of cancer screening among Latinos. In addition, research has shown that adherence to other preventive services increases adherence to CRC screening in Latinos (Gonzalez et al., 2012).

According to the BMHSU framework, an individual must perceive the need for and the possibility of illness in order for health care utilization to occur (Andersen and Newman, 1973). Need is generally measured by health-related quality of life (HRQOL). For example, although a lower HRQOL is related to increased chronic care-related utilization (Dominick et al., 2002), its relationship to the use of preventive services is unknown. In addition, Latinas with a family history of cancer show greater breast cancer screening use compared to those without family history, which relates to an increased awareness and perceived risk of breast cancer (Gorin and Heck, 2005; Aparicio-Ting and Ramirez, 2003; Cohen, 2006). Further, a previous cancer diagnosis has not been examined in relation to CRC screening.

The aim of this study was to examine factors associated with CRC screening use among US Latinos. In contrast to most of the studies that have examined multiple predictors of Latino CRC screening, this study was guided by a well-established theoretical framework (i.e., BMHSU). Use of health services can be conceptualized as discretionary or non-discretionary behavior (Andersen, 1968). The placement of and specified relationships among the predisposing, enabling, and need factors in the model varies depending on the character of the utilization variable (Andersen and Newman, 1973). Research shows that for the discretionary use of health services-such as preventive service utilization- predisposing (e.g., acculturation) and enabling (e.g., health insurance) factors may matter more than need (e.g., HRQOL) (Andersen, 1968). However, while predisposing factors do predict cancer screening among Latinos, the presence of enabling factors may counteract this role (Zambrana et al., 1999). We hypothesized that the enabling domains would most strongly predict CRC screening as compared to the predisposing and need domains.

## 2 Materials and methods

### 2.1 Participants and setting

The Hispanic Community Health Study/Study of Latinos (HCHS/SOL) is a multi-center epidemiologic study with the goal to assess the role of acculturation in the prevalence and development of disease, and to identify factors playing a protective or harmful role in the health of Hispanics/Latinos. The HCHS/SOL enrolled a cohort of Hispanic/Latino (i.e., from Cuban, Dominican, Puerto Rican, Mexican, Central and South American backgrounds) adults (n = 16,415) from four United States communities (Chicago, San Diego, Miami, and Bronx). Households were selected using a stratified two-stage probability sampling design and door-to-door recruitment from 2008 to 2011, and sampling weights were calibrated to the 2010 US Population Census (Sorlie et al., 2010). The HCHS/SOL Sociocultural Ancillary Study (SCAS) is a cross-sectional cohort study of 5313 adults representing multiple Hispanic/Latino background groups, recruited within nine months of their HCHS/SOL clinical baseline exam. The SCAS complemented the parent study with interview-administered sociocultural assessments. The SCAS sample is broadly representative of the HCHS/SOL cohort with the exception of lower participation in some high SES strata (Gallo et al., 2014). All SCAS participants with incomplete data on key variables (n = 152) or who were under the age of 50 years old (n = 2896) were excluded in analyses. Institutional Review Boards at all institutions reviewed and approved the research.

### 2.2 Measures

#### 2.2.1 CRC screening

Using data from the SCAS, three CRC screening behaviors were assessed: (A) CRC screening ever, (B) recent FOBT screening, and (C) recent sigmoidoscopy or colonoscopy. Recent CRC screening was assessed by several questions during the SCAS interview, including ever having an FOBT exam, time since last FOBT (< one year versus otherwise), ever having an endoscopic test (colonoscopy or sigmoidoscopy), and time since last endoscopic test (< 10 years versus otherwise). Sigmoidoscopy and colonoscopy in last 10 years were grouped together because items stemmed from the 2008 National Health Interview Survey (NHIS) which assessed both sigmoidoscopy and colonoscopy screening procedures in one item (Centers for Disease Control and Prevention NCfHS, 2008).

#### 2.2.2 Predisposing domain measures

The analysis sample was restricted to those aged 50–75 to correspond with current CRC screening guidelines and those in place at the time of the survey. Age was assessed by self-report date of both and sex was assessed by self-report. Education was coded into categories (< high school, high school, and ≥ high school) (Centers for Disease Control and Prevention, 2006), annual household income was coded into three categories (< $30,000, > $30,001, and unknown). Three country of birth categories (i.e., born in 50 US states and territories, foreign-born in the US for <10 years, and foreign born in the US for ≥10 years) were created. Language-based acculturation was assessed by survey preference (i.e., English versus Spanish). Hispanic/Latino Heritage was self-reported and the following categories were created: Dominican, Central/South American, Cuban, Mexican, Puerto Rican, and Other/more than one).

#### 2.2.3 Enabling domain measures

Health insurance, recent physician visit, and recent mammography (for women only) were self-reported in the parent study and items were derived from the Behavioral Risk Factor Surveillance System (Centers for Disease Control and Prevention, 2006). Insurance was assessed by self-report (being currently insured versus none). Recent physician visit was assessed by time since last doctor visit for a routine checkup; a binary variable was created (visit in the past year versus otherwise). In addition, for women only, mammography screening was assessed and recoded as (< one year versus otherwise to correspond to Agency for Health Research and Quality(AHRQ)/National Cancer Institute (NCI) screening guidelines (Agency for Healthcare Research and Quality (AHRQ), 2012; National Cancer Institute, 2013) for women aged 50 to 75 years old).

#### 2.2.4 Need-related domain measures

Need was measured by the health-related quality of life (HRQOL) and personal and family history of cancer that were self-reported. For history of cancer, dichotomous variables were created (family or personal history of cancer versus otherwise). HRQOL was assessed with the mental and physical health component scores from the SF-12 Health Survey Version 2, (SF12v2) under license by QualityMetric (Ware et al., 2002). The scales have demonstrated good test–retest reliability in national studies and have been validated in Hispanic/Latinos (Gandek et al., 1998; Ware et al., 1998). To compare to population norms, mental and physical health sub-scale scores were *Z*-transformed, and scaled to a mean of 50 and standard deviation of 10, ranging from 0 to 100. A higher score indicated greater HRQOL.

### 2.3 Statistical analyses

All analyses were conducted using procedures in SAS version 9.2 to incorporate the complex sampling design and the sampling weights. Based on prior studies using the BMHSU, (Miller et al., 2008; Fernandez and Morales, 2007; Gorin and Heck, 2005), this study used logistic regression models to test the direct effects of the three BMHSU domains [i.e., predisposing, enabling, and need] on CRC screening (Hosmer and Lemeshow, 2000) [i.e., CRC screening ever, FOBT in last year, or endoscopic test (colonoscopy/sigmoidoscopy) in last 10 years]. Each multivariable model included all potential correlates that represented the three BMHSU domains, accounting for the sampling and stratified by gender. To convey the magnitude and direction of the significance of each independent variable, odds ratios, 95% confidence intervals (CIs), and statistical significance values were calculated (Kleinbaum et al., 2007; Gardner and Altman, 1986). All assumptions of logistic regression were met (Menard, 2002). Results were stratified by sex to allow for the examination of the impact of recent breast cancer screening on CRC screening among women only.

## 3 Results

### 3.1 Sample characteristics

Participant predisposing, enabling, and need characteristics and frequency of CRC screening are shown in Table 1 by sex. Around two-thirds (63%) were female. Most were born outside the US or territories (78% of females; 77% of males), and most preferred completing the questionnaires in Spanish (87% of females; 85% of males). Both sexes had an average age of 60 years. Males were more likely to report an annual household income of greater than $30,000 (20% of females and 28% of males) (p < .01), and less than half of both sexes (42% of females and 41% of males) did not graduate from high school. Across both sexes, most were Cuban (27% females, 34% males) and Mexican (29% females, 25% males), followed by Puerto-Rican (20% females, 19% males), Central/South American (13% females, 12% males), Dominican (10% females, 9% males), and Other/ more than one Hispanic/Latino background group (1% females and males).

**Table 1:** Frequency of predisposing, enabling, and need characteristics for colorectal cancer screening among Latinos by sex (aged 50–75), N = 2265.

	Female % (SE) (n = 1422)	Male % (SE) (n = 843)	p value
Predisposing domain^a^			
Age (years), M(SE)	59.5 (0.4)	59.7 (0.3)	0.727
Education			0.890
Less than high school	42.3 (2.6)	41.4 (2.6)	
High school	18.8 (1.4)	18.3 (1.6)	
High school or greater	38.9 (2.4)	40.3 (2.3)	
Annual household income			<0.001
<$30,000	71.4 (2.4)	69.2 (2.5)	
≥$30,000	20.4 (2.4)	27.8 (2.4)	
Not reported	8.1 (1.1)	2.9 (0.7)	
Language of interview			0.598
English	13.0 (2.5)	14.6 (2.1)	
Spanish	87.0 (2.5)	85.4 (2.1)	
Country of birth^a^			0.523
U.S.	22.1 (2.5)	22.9 (2.3)	
Foreign-born <10 years	22.3 (2.3)	19.3 (2.0)	
Foreign-born ≥10 years	55.4 (2.6)	57.7 (2.4)	
Hispanic/Latino background			0.1979
Dominican	9.9(1.2)	8.7(1.31)	
Central/South American	12.6(1.1)	11.6(1.3)	
Cuban	27.4(2.7)	33.7(3.2)	
Mexican	29.3(2.8)	25.3(2.5)	
Puerto-Rican	19.7(2.5)	19.2(2.1)	
Other/more than one	0.9(0.2)	1.2(0.4)	
Enabling domain			
Recent physician visit (<1 year)	82.6 (1.7)	75.3 (2.0)	0.002
Health insurance	60.3 (2.6)	61.0 (2.5)	0.849
Breast cancer screening adherence^b^	56.9 (2.6)	–	–
Need domain			
Physical health status, M(SE)	45.6 (0.4)	46.8 (0.6)	<0.001
Mental health status, M(SE)	46.9 (0.8)	50.7 (0.5)	<0.001
Family history of cancer	36.5 (2.0)	27.3 (2.2)	0.002
Personal history of cancer	5.8 (0.9)	6.5 (1.3)	0.667
CRC screening			
Any colorectal exam ever	62.2 (2.3)	59.8 (2.4)	0.397
Colonoscopy ever	44.5 (2.7)	35.6 (2.6)	0.008
Sigmoidoscopy ever	8.8 (2.0)	7.2 (1.1)	0.4568
Sigmoidoscopy/colonoscopy last 10 years	45.4 (2.7)	37.3 (2.6)	0.0141
Fecal Occult Blood Test (FOBT) ever	46.4 (2.6)	49.2 (2.4)	0.3744
FOBT last year	23.9 (2.7)	27.1 (2.1)	0.3585

Note. All values were weighted for survey design and non-response.

a US-born includes 50 states or US territories.

b Defined as mammography last year.

Females were more likely to have a physician visit in the last year (83% of females and 75% of males) (p < .01), while more than half were insured (60% of females and 61% of males), and more than half of females (57%) had a mammogram in the last year.

Males (M = 46.8, SE = 0.6) reported significantly higher physical health status than females (M = 45.6, SE = 0.4) (p < .01) and males reported greater mental health status (M = 50.7, SE = 0.5) than females (M = 46.9, SE = 0.8). More females (37%) than males (27%) reported a family history of any type of cancer (p < .01) and few reported (6% of females and 7% of males) a personal history of cancer. Results showed that 62% of females and 60% of males had ever had a CRC exam. Females were more likely to have had a colonoscopy ever (45%) compared to males (36%) (p < .01), and a sigmoidoscopy/colonoscopy in the last 10 years (45% of females, 37% of males) (p < .05). Less than half reported ever having a FOBT test (46% of females and 49% of males), with around one-fourth (24% of females and 27% of males) reporting a FOBT test in the last year.

### 3.2 Correlates of CRC screening: females

In adjusted analyses, preferring Spanish language compared to English language (*OR*, 0.37; 95% CI, 0.17–0.80) was associated with a lower likelihood of ever having had a CRC screening exam (p ≤ .05). In addition, being foreign born and residing in the US for 10 or more years compared to US born (*OR*, 2.64, 95% CI, 1.04–6.67), having a physician visit in the past year (*OR*, 2.06; 95% CI, 1.26–3.35), a mammogram in the last year (*OR*, 2.06; 95% CI, 1.36–3.13), and having a personal history of cancer (*OR*, 1.92; 95% CI, 1.06–3.46) significantly predicted having any CRC screening exam ever (p ≤ .05); all other variables were not significant (**Model 1**, Table 2).

**Table 2:** Multivariable logistic regression models of CRC screening^a^, for Latina females ages 50–75, N = 1422.

Behavioral Model of Health Services Utilization	Model 1 CRC Screening ever	Model 2 FOBT last year	Model 3 Sig/Col test < 10 years
	OR (95% C.I.)	OR (95% C.I.)	OR (95% C.I.)
Predisposing domain			
Age, yrs	1.03(1.01, 1.06)⁎	1.00 (0.96, 1.04)	1.03(0.99, 1.06)
Education			
< High school^b^	1.00	1.00	1.00
High school	0.81(0.49, 1.33)	0.96(0.52, 1.76)	0.61(0.34, 1.05)
≥ High school	1.06(0.67, 1.66)	1.00(0.52, 1.90)	0.78(0.46, 1.32)
Annual household income			
< 30,000^b^	1.00	1.00	1.00
≥ 30,000	1.64(0.96, 2.81)	1.20(0.58, 2.52)	1.18(0.63, 2.26)
Unknown	1.38(0.70, 2.71)	1.02(0.47, 2.44)	0.99(0.48, 2.05)
Language			
English b	1.00	1.00	1.00
Spanish	0.37(0.17, 0.80)⁎	0.93(0.35, 2.44)	0.56(0.24, 1.27)
Country of birth			
U.S.^b^, ^c^	1.00	1.00	1.00
Foreign-born <10 years	1.77(0.64, 4.75)	2.04(0.73, 5.68)	0.81(0.28, 2.37)
Foreign-born ≥10 years	2.64(1.04, 6.67)⁎⁎	2.313(0.88, 5.17)	1.30(0.48, 3.49)
Hispanic background			
Dominican	1.35(0.72, 2.52)	0.53(0.27, 1.06)	1.83(0.93, 3.56)
Central/South American	1.22(0.71, 2.11)	0.90(0.54, 1.50)	1.51(0.87, 2.60)
Cuban	0.98(0.56, 1.69)	0.74(0.41, 1.33)	0.85(0.47, 1.53)
Mexican	1.00	1.00	1.00
Puerto-Rican	3.43(1.40, 8.42)	2.13(0.86, 5.28)	2.72(1.13, 6.54)
Other/more than one	3.20(0.93, 10.99)	0.32(0.07, 1.43)	2.78(0.60, 12.72)
Enabling domain			
Health insurance			
No^b^	1.00	1.00	1.00
Yes	1.44(0.94, 2.21)	2.20(1.30, 3.75)⁎⁎	1.89(1.17, 3.05)⁎⁎
Recent physician visit			
No^b^	1.00	1.00	1.00
Yes	2.06(1.26, 3.35)⁎⁎	3.57(1.65, 7.71)⁎⁎	2.35(1.19, 4.53)⁎
Breast cancer screening adherence^d^			
No^b^	1.00	1.00	1.00
Yes	2.06(1.36, 3.13)⁎⁎⁎	1.42(0.78, 2.56)	1.90(1.12, 3.18)⁎
Need domain			
Physical health status	0.99(0.98, 1.01)	1.01(0.99, 1.02)	1.001(0.98, 1.01)
Mental health status	1.01(0.98, 1.02)	1.02(0.99, 1.04)	1.005(0.98, 1.02)
Family history of cancer			
No^b^	1.00	1.00	1.00
Yes	1.26(0.90, 1.81)	0.70(0.43, 1.08)	1.36(0.93, 2.00)
Personal history of cancer			
No^b^	1.00	1.00	1.00
Yes	1.92(1.06, 3.46)⁎	1.10(0.52, 2.36)	1.07(0.57, 2.04)

All models were weighted for survey design and non-response.

a Three separate DVs were examined: (A) CRC screening ever (B) recent FOBT screening <1 year and (C) recent sigmoidoscopy/colonoscopy <10 years.

b Reference category.

c US-born includes 50 states or US territories.

d Defined as mammography last year.

⁎⁎⁎ p < .001.

⁎⁎ p < .01.

⁎ p < .05.

In adjusted analyses, having health insurance (*OR*, 2.20; 95% CI, 1.30–3.75) and a physician visit in the past year (*OR*, 3.57; 95% CI, 1.65–7.71) significantly predicted having any a FOBT test in the last year (p ≤ .05); all other variables were not significant (**Model 2**, Table 2). In adjusted analyses, having health insurance (*OR*, 1.89; 95% CI, 1.17–3.05), a physician visit in the past year (*OR*, 2.35; 95% CI, 1.19–4.53), and having a mammogram in the last year (*OR*, 1.90; 95% CI, 1.12–3.18) significantly predicted having an endoscopic exam in the last 10 years (p ≤ .05); all other variables were not significant (**Model 3**, Table 2).

### 3.3 Correlates of CRC screening: males

In adjusted analyses, having a Dominican background versus Mexican (*OR*, 2.38; 95% CI, 1.04–5.46), having health insurance (*OR*, 2.78, 95% CI, 1.76–4.40), and a physician visit in the past year (*OR*, 2.23; 95% CI, 1.30–3.80) significantly predicted having any CRC screening exam ever (p ≤ .05); all other variables were not significant (**Model 1**, Table 3).

**Table 3:** Multivariable logistic regression models of CRC screening^a^, for Latino males ages 50–75, N = 843.

Behavioral Model of Health Services Utilization	Model 1 CRC screening ever	Model 2 FOBT last year	Model 3 Sig/Col test < 10 years
	OR (95% C.I.)	OR (95% C.I.)	OR (95% C.I.)
Predisposing domain			
Age, yrs	1.02(0.99, 1.06)	0.99(0.96, 1.03)	1.02(0.99, 1.06)
Education			
< High school^b^	1.00	1.00	1.00
High school	1.03(0.64, 1.64)	0.80(0.46, 1.39)	1.45(0.80, 2.64)
≥ High school	1.15(0.70, 1.9)	0.76(0.47, 1.24)	1.24(0.72, 2.14)
Annual household income			
< 30,000^b^	1.00	1.00	1.00
≥ 30,000	1.18(0.71, 1.98)	1.23(0.78, 1.95)	1.30(0.75, 2.26)
Unknown	1.18(0.45, 3.13)	0.82(0.26, 2.55)	0.53(0.19, 1.46)
Language			
English^b^	1.00	1.00	1.00
Spanish	0.90(0.43, 1.83)	1.07(0.53, 2.16)	0.95(0.48, 1.87)
Country of birth			
U.S.^b^, ^c^	1.00	1.00	1.00
Foreign-born <10 years	0.51(0.18, 1.43)	0.67(0.23 1.96)	0.92(0.48, 3.56)
Foreign-born ≥10 years	0.74(0.31, 1.80)	0.81(0.33, 2.01)	1.48(0.42, 5.11)
Hispanic/Latino Background			
Dominican	2.38 (1.04, 5.46)⁎	1.30 (0.63, 2.68)	3.14(1.47, 6.74)
Central/South American	0.94 (0.49, 1.79)	0.79 (0.37, 1.58)	1.39(0.71, 2.71)
Cuban	0.55 (0.30, 1.005)	0.97 (0.51, 1.83)	0.63(0.33, 1.19)
Mexican	1.00	1.00	1.00
Puerto-Rican	1.15 (0.49, 2.69)	1.26 (0.50, 3.18)	2.75(0.78, 9.68)
Other/more than one	1.72 (0.34, 8.49)	<0.001 (<0.001, <0.001)	3.69(0.76, 17.96)
Enabling domain			
Health insurance			
No^b^	1.00	1.00	1.00
Yes	2.78 (1.76, 4.40)⁎⁎⁎	2.93 (1.73, 5.14) ⁎⁎⁎	2.33 (1.49, 3.66)⁎⁎⁎
Recent physician visit			
No^b^	1.00	1.00	1.00
Yes	2.23 (1.30, 3.80)⁎⁎	1.84(0.90, 3.75)	5.38 (2.77, 10.45)⁎⁎⁎
Need domain			
Physical health status	1.005 (0.98, 1.02)	1.01 (0.99, 1.03)	1.00 (0.97, 1.02)
Mental health status	1.005 (0.99, 1.02)	1.01 (0.99, 1.03)	1.007 (0.98, 1.02)
Family history of cancer			
No^b^	1.00	1.00	1.00
Yes	1.10 (0.72, 1.67)	0.78 (0.47, 1.30)	1.24 (0.76, 2.00)
Personal history of cancer			
No^b^	1.00	1.00	1.00
Yes	2.36 (0.71, 7.77)	0.70(0.26, 1.88)	3.25 (1.13, 9.35)⁎

All models were weighted for survey design and non-response.

a Three separate DVs were examined: (A) CRC screening ever (B) recent FOBT screening <1 year and (C) recent sigmoidoscopy/colonoscopy <10 years.

b Reference category.

c US-born includes 50 states or US territories.

⁎⁎⁎ p < .001.

⁎⁎ p < .01.

⁎ p < .05.

In adjusted analyses, having health insurance (*OR*, 2.93; 95% CI, 1.73–5.14) significantly predicted having a FOBT test in the last year (p ≤ .05); all other variables were not significant (**Model 2**, Table 3). In adjusted analyses, having health insurance (*OR*, 2.33; 95% CI, 1.49–3.66) a physician visit in the past year (*OR*, 5.38; 95% CI, 2.77–10.45), and a personal history of cancer (*OR*, 3.25, 95% CI, 1.13–9.35) significantly predicted having an endoscopic exam in the last 10 years (p ≤ .05); all other variables were not significant (**Model 3**, Table 3).

## 4 Discussion

This study found that for both men and women, common factors associated with all three CRC screening indicators (i.e., CRC screening ever, FOBT last year, endoscopic exam last 10 years) were enabling factors, including having health insurance, having a physician visit in the last year, and having a recent mammogram (for women only). These results concur with previous regional (Fernandez and Morales, 2007; Gonzalez et al., 2012; Castaneda et al., 2014; Castaneda et al., 2012; Savas et al., 2015) and national studies (i.e., using 2000 NHIS (Gorin and Heck, 2005) and 2000–2007 Medical Expenditure Panel Survey data (Miranda et al., 2012)), which have shown that insurance and recent physician visit, and prior mammography (all enabling variables) are consistently the strongest predictors of breast, cervical, and CRC screening among Latinos.

In addition, for both men and women, a personal history of cancer (i.e., being a cancer survivor) was related to CRC screening. Men with a personal history of cancer were more likely to have received an endoscopic exam in the last 10 years. Among women, those with a personal history of cancer were more likely to have ever had a CRC screening test. Research has shown that personal or family history of cancer is a significant correlate of Latina women's breast cancer screening, relating to increased awareness of the disease (Gorin and Heck, 2005; Cohen, 2006). In our study, only a personal history, and not a family history of cancer, was significantly related to CRC screening.

Men of Dominican heritage were more likely than those of Mexican heritage to have ever received a CRC screening or have had an FOBT in the last year. Given that national (e.g., MEPS) and regional studies (Fernandez and Morales, 2007; Gonzalez et al., 2012; Castaneda et al., 2014; Castaneda et al., 2012; Savas et al., 2015) have mostly focused on Mexican background populations, more research is needed to understand community contextual factors that may be contribute to these differences.

For women, results from this study showed that being more acculturated (i.e., prefer English language or foreign born but have lived in the US for 10 or more years) was significantly associated with having ever had a CRC screening. Being more acculturated is a correlate that has been shown to be associated with Latino cancer screening in other regional studies in Texas (Fernandez and Morales, 2007) and Michigan (Castaneda et al., 2012), while in other areas, such as California, a lower acculturation has been shown to be related to breast cancer screening among Latinas (Castaneda et al., 2014). These findings suggest a need to understand the community context in which diverse Hispanics/Latinos reside (e.g., racial/ethnic diversity, socioeconomic status, health care access), and language/cultural concordance of staff and health care providers which may explain differences in screening rates.

Given that this study (2008–2011) occurred prior to the implementation of the Affordable Care Act, which was signed into law in 2010, it can be hypothesized that this effect of the enabling variables on CRC screening may be attenuated going forward into the future. However, a recent study that examined the impact of the Affordable Care Act on Latino health care access and utilization showed that from 2011 to 2015, while overall access and utilization increased among some Latinos, some groups, such as noncitizens and non-English speakers continue to have limited access to care (Alcala et al., 2017). It is possible these groups may also experience lower access to regular cancer screening and increased cancer health disparities, including later stage of diagnosis, and more advanced disease due to fewer treatment options.

There are several factors in this study that may limit interpretation of results. First, the cross-sectional data collected on CRC screening were based on self-report. Second, additional contextual information about the nature and accessibility of the type of healthcare available was not collected for HCHS/SOL participants. Third, this study is limited to four major metropolitan cities in the US and does not include rural Hispanics/Latinos. However, the US 2010 Census data reveal that 80.7% of the US population resides in urban (versus 19.3% rural) areas (US Census Bureau, 2013). It is estimated that 90% or more of Hispanics/Latinos in the US live in metropolitan areas (Mather and Pollard, 2014; Guzman, 2001). Given that the four field centers in this study are located within the top 11 ranked metropolitan areas (PewResearch, 2014a) and the top ten counties (PewResearch, 2014b) in the US for number of Hispanics/Latinos, results from this study, although not representative of all Latinos in the US, are representative for each of these large concentration of Hispanic/Latino backgrounds in metropolitan areas.

## 5 Conclusion

In conclusion, results showed that approximately 60% of women and men aged 50 to 75 years had any CRC exam ever. These rates are significantly lower than the Healthy People 2020 and American Cancer Society goal of 80% screening. Around one fourth had a FOBT in the last year (27% of males, 24% of females) in this study, which is higher than previous 2015 NHIS data (7%) for Hispanics/Latinos (American Cancer Society, 2017). Similar to previous regional and national studies of Latino cancer screening that have been theoretically guided by the BMHSU (Fernandez and Morales, 2007; Gorin and Heck, 2005; Gonzalez et al., 2012; Castaneda et al., 2014; Castaneda et al., 2012; Savas et al., 2015), enabling domain variables (i.e., health insurance, health care utilization) consistently predicted CRC screening for all three CRC screening indicators in this study. Our findings suggest that improved access to care among Latinos is one key to reaching higher CRC screening rates in all US communities.

## Declaration of Competing Interest

The authors declare there is no conflict of interest.
